# Editorial: Neurodegeneration and Neuroprotection in Retinal Disease, Volume II

**DOI:** 10.3389/fnins.2022.1009228

**Published:** 2022-08-23

**Authors:** Giovanni Casini, Mohammad Shamsul Ola, Peter Koulen

**Affiliations:** ^1^Department of Biology, University of Pisa, Pisa, Italy; ^2^Department of Biochemistry, College of Science, King Saud University, Riyadh, Saudi Arabia; ^3^Department of Ophthalmology, Vision Research Center, School of Medicine, University of Missouri–Kansas City, Kansas City, MO, United States; ^4^Department of Biomedical Sciences, School of Medicine, University of Missouri–Kansas City, Kansas City, MO, United States

**Keywords:** oxidative stress, inflammation, apoptosis, nutraceuticals, drug delivery

The goal of the Frontiers Research Topic, *Neurodegeneration and Neuroprotection in Retinal Disease, Volume II*, was to expand on Volume I of this Research Topic. Specifically, the Research Topic aimed to *elucidate (i) pathophysiological mechanisms of neurodegeneration in retinal diseases; (ii) new neuroprotective substances, with a particular attention to nutraceuticals, that may be used to treat retinal pathologies; (iii) new methods of neuroprotective drug delivery to the retina*.

Diseases impairing retina function continue to generate an increasing healthcare burden across the globe affecting both patients and their families. As a majority of these diseases produces vision loss and blindness by impairing the viability and ultimately inducing cell death of retinal neurons, mechanisms underlying retinal neurodegeneration remain only partially identified. While the pathophysiology of glaucoma, diabetic retinopathy (DR), age-related macular degeneration (AMD), and retinitis pigmentosa differ widely, all have degeneration of the neural retina in common. Therefore, common features of neurodegeneration leading ultimately to the loss of neurons and visual function, can be not only determined, but can also lead to insights into its molecular, cellular and systemic mechanisms. The growing field of neuroprotection utilizes these novel insights into the causes of neurodegeneration to devise both preventative and therapeutic strategies covering areas ranging from traditional nutraceutical and pharmaceutical approaches to groundbreaking new cell-based and personalized medicine therapies. Due to the complex, chronic and multifactorial nature of neurodegeneration in retinal pathologies research efforts continue and increase to innovate and improve impactful *in vitro* and *in vivo* models. Innovation of such preclinical research tools remains a critical component of research benefiting both the neurodegeneration and neuroprotection fields and accelerating and strengthening the critical interaction between both of these fields.

The Frontiers Research Topic, “*Neurodegeneration and Neuroprotection in Retinal Disease, Volume II*,” contributes to this broad range of complementary research efforts with two reviews and seven research articles:

Aldosari et al. provide a systematic review in the field of DR, specifically focusing on a synthesis of recent advances in preclinical and clinical studies on the role of metabolites in neurovascular damage contributing to neurodegeneration in DR. Reviewing key signaling pathways affecting or affected by sugar, lipid, and amino acid metabolites of carbohydrate, lipid, and amino acids the authors integrate key aspects of endocrinology, immunology and ophthalmology research including underlying seminal basic science research. At the same time, the authors also identify challenges and opportunities, where clinical phenotypes obtained through metabolic profiling have not been linked with mechanisms underlying pathophysiology and where new research tools to identify mechanisms underlying metabolic defects have become available, respectively (Aldosari et al.).

Bridging the fields of nutraceuticals, dietary supplements and small molecule therapeutics as they relate to neuroprotection, Edwards et al. provide a review of molecular mechanisms underlying the therapeutic actions of vitamin E in AMD. Starting with successes and shortcomings in the clinical use of vitamin E to control retinal neurodegeneration and other medical conditions, the authors integrate recent mechanistic findings from the preclinical and clinical scientific literature to come full circle and provide conclusions and directions for research to improve future clinical intervention approaches for AMD and potentially other retinal diseases (Edwards et al.).

Three of the research articles advance research tools and novel approaches to identify mechanisms of neurodegeneration geared toward improved neuroprotective strategies:

Rohowetz et al. explore the impact of abnormalities of the anterior segment on ocular hypertension in a widely used mouse model of glaucoma, the DBA/2J mouse, and in DBA/2J-Gpnmb+/SjJ control mice. They found that while common in both the glaucomatous and the control mouse strains, corneal calcification does not contribute to the development of an elevated intraocular pressure in DBA/2J mice, while iris pigment dispersion does. These findings provide important insights in the use of and for the interpretation of data obtained from this key preclinical glaucoma model (Rohowetz et al.).

Gajendran et al. present a novel strategy to identify and analyze changes in electroretinography signals resulting from glaucoma. Using a machine-learning algorithm, they were able to identify functional changes and deficits in a mouse model of glaucoma and conclude that the novel tool can potentially facilitate the quantitative and non-invasive assessment of both early-stage glaucoma and the success of therapeutic intervention (Gajendran et al.).

Asatryan et al. describe a new human retinal pigment epithelial cell line as an innovative tool to conduct research on a cell type critical for retina and particularly photoreceptor cell function and health. Gene expression profiling, and developmental, structural, functional and pharmacological characterization of these cells lead the authors to conclude that the new cell line will be able to contribute significantly to the field of neuroprotection research (Asatryan et al..

Four of the research articles report new mechanisms of action representing clinically relevant targets for neuroprotective strategies:

Nath and Fort identify distinct molecular signaling pathways underlying αA-crystallin-mediated neuroprotection. The authors describe neuroprotective activity of αA-crystallin independent of well characterized neuroprotective kinase signaling pathways and leading to the identification of new potential neuroprotective approaches (Nath and Fort).

Piano et al. measure the neuroprotective potential of the nutraceuticals naringenin and quercetin in a mouse model of retinal degeneration. The authors find robust protection from photoreceptor cell degeneration mediated by antioxidant and anti-apoptotic properties of the nutraceutical molecules suggesting potential efficacy as nutraceutical therapy for retinitis pigmentosa patients (Piano et al.).

Wei et al. identify sirtuin 4 (SIRT4) in Müller glia of the retina and describe how activation of SIRT4 can be used to increase glutamine synthetase expression and thereby neuroprotection. The authors conclude that SIRT4 can potentially become a target for long-term pharmacotherapy of retinal pathologies (Wei et al.).

Fedotkina et al. determine genetic risk factors for proliferative diabetic retinopathy resulting from exposure to starvation and observe changes in retina development resulting from glucose starvation. The authors suggest opportunities for neuroprotective intervention with respect to both developmental stage and potential molecular targets (Fedotkina et al.).

In summary, together with Volume I, this Volume II of the Frontiers Research Topic on Neurodegeneration and Neuroprotection in Retinal Disease constitutes a valid contribution to the understanding of the neurodegeneration phenomena in retinal diseases and to the possible design of new therapeutic approaches that could limit the socio-economic burden of sight-threatening pathologies.

## Author contributions

PK conceived and wrote the editorial. All authors edited and reviewed the manuscript. All authors contributed to the article and approved the submitted version.

## Funding

This publication was supported by the Felix and Carmen Sabates Missouri Endowed Chair in Vision Research.

## Conflict of interest

The authors declare that the editorial was written in the absence of any commercial or financial relationships that could be construed as a potential conflict of interest.

## Publisher's note

All claims expressed in this article are solely those of the authors and do not necessarily represent those of their affiliated organizations, or those of the publisher, the editors and the reviewers. Any product that may be evaluated in this article, or claim that may be made by its manufacturer, is not guaranteed or endorsed by the publisher.

